# Squamous cell lung carcinoma with gastrointestinal metastasis: a case report and review of literature

**DOI:** 10.3389/fonc.2023.1138871

**Published:** 2023-04-21

**Authors:** Feifei Li, Yiqiang Liu, Ke Xu, Quan Yao, Qiang Li, Hong Wu

**Affiliations:** ^1^ Sichuan Cancer Hospital & Institute Sichuan Cancer Center, School of Medicine University of Electronic Science and Technology, Chengdu, Sichuan, China; ^2^ Collaborative Innovation Centre of Regenerative Medicine and Medical BioResource Development and Application Co-constructed by the Province and Ministry, Guangxi Medical University, Nanning, Guangxi, China; ^3^ Department of Oncology & Cancer Institute, Sichuan Academy of Medical Sciences, Sichuan Provincial People’s Hospital, University of Electronic Science and Technology of China, Chengdu, Sichuan, China; ^4^ Department of Laboratory Medicine and Sichuan Provincial Key Laboratory for Human Disease Gene Study, Sichuan Provincial People’s Hospital, University of Electronic Science and Technology of China, Chengdu, Sichuan, China

**Keywords:** squamous cell lung carcinoma (LUSC), gastrointestinal metastasis, rare cecum metastasis, surgical resection, neoadjuvant chemoradiotherapy

## Abstract

Squamous cell lung carcinoma (LUSC) originates from squamous cells and has a high rate of metastasis and recurrence. The lack of effective genetic targets and specific therapies has resulted in a poor prognosis for patients with LUSC. Gastrointestinal metastasis of LUSC is a rare occurrence in clinical practice. Patients with gastrointestinal metastasis usually have worse overall survival and the process of diagnosis is more complicated than those with metastasis elsewhere. What’s more, there are no helpful guidelines for treating patients with a clinically confirmed diagnosis of gastrointestinal metastasis, which means the treatment method is limited. Here, we review the clinical features, diagnosis, and treatment of LUSC patients with gastrointestinal metastasis and report a rare case of LUSC accompanied by gastrointestinal metastasis. The patient was admitted to the hospital with coughing and hemoptysis. A tumor was found in his lung, and lesions were initially controlled with standard treatment. The patient’s tumor re-occurred again shortly for which treatment was lacking. Without effective treatment methods, the disease was difficult to control. Our learnings from the case demonstrate that LUSC metastasizes to secondary lymphoid organs of the gastrointestinal tract, usually with a poor prognosis.

## Introduction

Squamous cell lung carcinoma (LUSC) is a prevalent type of non-small cell lung cancer (NSCLC), accounting for approximately 25% to 30% of all NSCLCs. Epidemiological investigations have shown that LUSC occurs more commonly in elderly men and is more strongly associated with smoking than any other type of NSCLC (1, 2). The early symptoms of LUSC are mild and easy to ignore. At the time of diagnosis, most patients are already in the advanced stage of the disease and often present with distal metastasis, which results in a poor prognosis of advanced LUSC with a 5-year survival rate of only 6% (3).

In NSCLC, metastases occur easily and are more often localized to the bone (34%), liver (20%), brain, adrenal glands, thoracic cavity, and lymph nodes (4, 5). Some rare sites of metastasis include the soft tissues (0-0.8%), bone marrow (0.16%), intestine (0.2%–1.8%), eye (0.1%–7%), thyroid (1.6%), tongue (0.2%–1.6%), pancreas, spleen, peritoneum, ovary, heart, breast, kidney, tonsil, and nasal cavity (6–19). Compared to other types of NSCLC, LUSC has a higher rate of gastrointestinal metastasis, which has been associated with a worse outcome (9, 20–22). Clinically diagnosing the gastrointestinal metastasis of LUSC mainly depends on imaging examination results (6). There are no helpful guidelines for treating patients with a clinically confirmed diagnosis of gastrointestinal metastasis of LUSC. Here, we review the clinical features, diagnosis, and treatment of LUSC patients with gastrointestinal metastasis and report a rare case of LUSC accompanied by gastrointestinal metastasis.

## Case presentation

**Figure 1:**
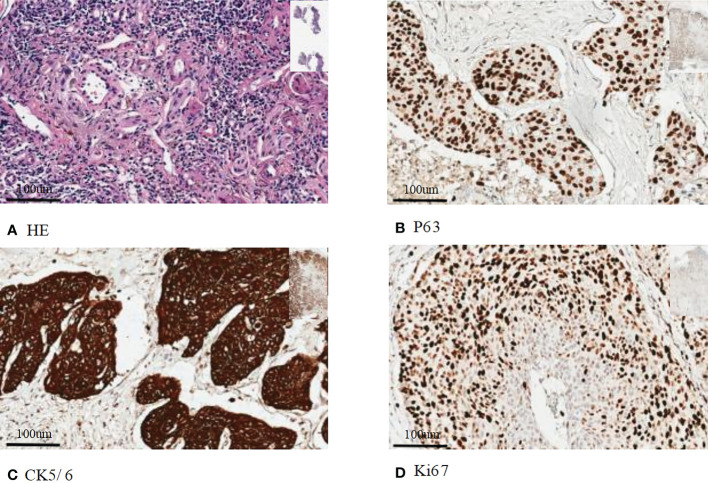
Immunohistochemical staining of histopathology: **(A)** Hematoxylin eosin staining of primary squamous cell carcinoma; **(B)** Immunohistochemical staining of P63; **(C)** Immunohistochemical staining of CK5/6; **(D)** Immunohistochemical staining of Ki67. Bar=100 um.

**Figure 2:**
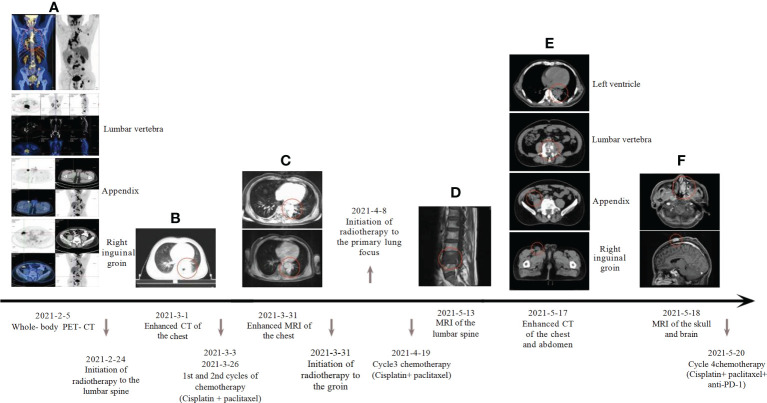
Systemic treatment strategies: **(A)** Whole-body PET-CT: maximum standardized uptake value: lung tumor 10.6, cecum 11.2, lumbar vertebra 15.7, inguinal groin 10.7, left ventricle 9.0, Cranial top skin 18.6; **(B)** Baseline computed tomography-enhanced CT of the chest; **(C)** The patient’s chest enhancement MRI was reassessed two weeks after chemotherapy; **(D)** MRI of lumbar spine metastases; **(E)** Review of chest, abdomen and pelvic enhancement CT; **(F)** MRI of the head suggests nasal and scalp neoplasm.

**Figure 3:**
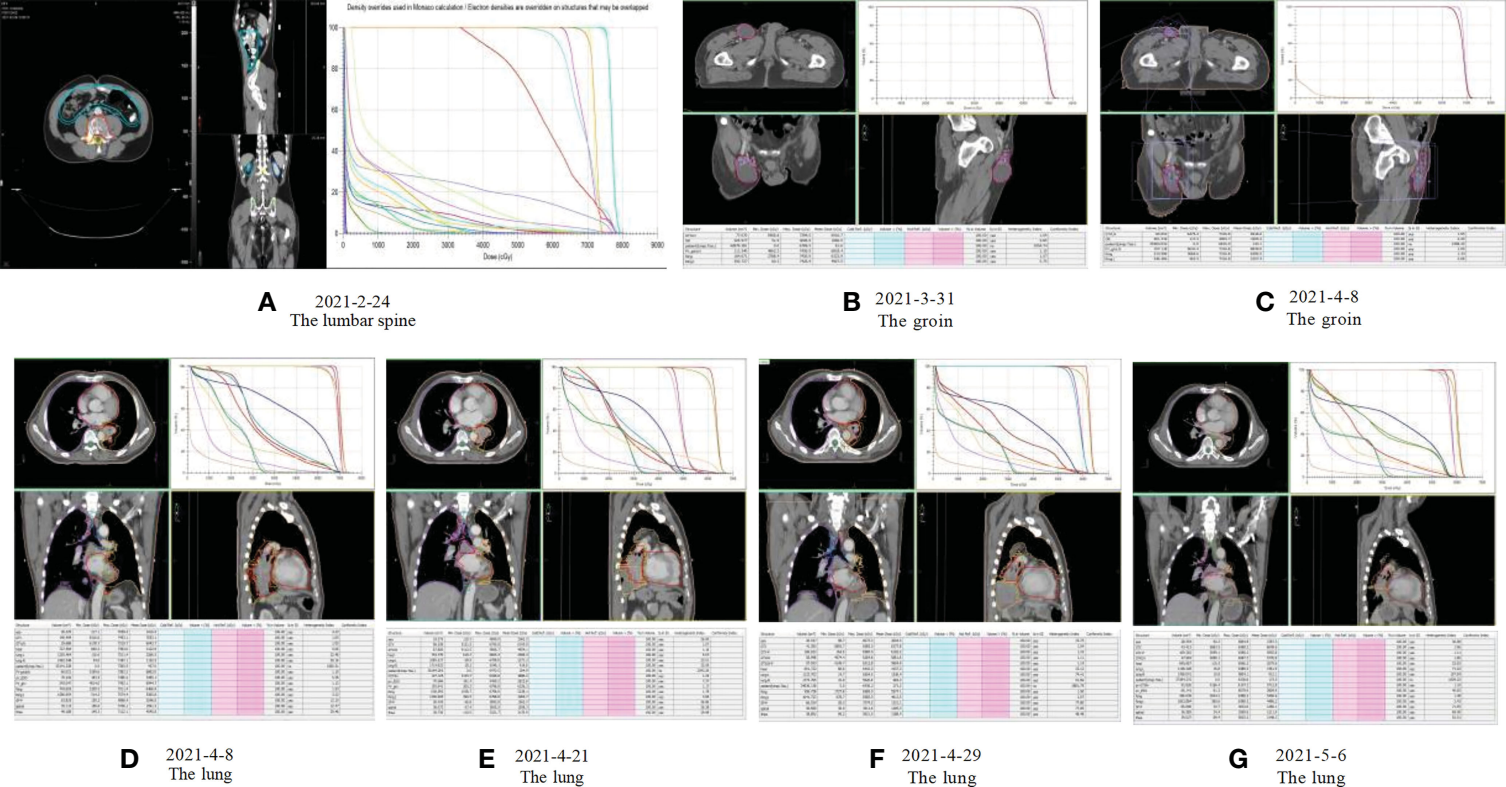
Radiotherapy plan: **(A)** Radiotherapy plan for the lumbar spine: Gross tumor volume (GTV L4-5) 35Gy/10F, Clinical target volume (CTV) 30Gy/10F; **(B, C)** Radiotherapy plan for inguinal metastatic lymph nodes: Gross tumor volume (GTVp) 52Gy/23F; **(D-G)** Radiotherapy plan for primary lung lesions: Gross tumor volume (GTVp) 47.6Gy/19F, Lymph node target volume (GTVn) 41.8Gy/19F.

Our patient is a 50-year-old male who presented with lumbago, cough, and sputum accompanied by hemoptysis and was admitted to our outpatient clinic. Pathological biopsy confirmed squamous cell carcinoma of the left lower lung lobe with CK5/6 (+) and P63 (+). Similar pathological results confirmed cecum metastasis (**Figure 1**). FDG-PET CT identified an intensely avid lobe of left lung (LLL) mass (maximum standardized uptake value 10.6 (Max SUV), 32 mm×30 mm) extending to the cecum, top cranial skin, lumbar vertebra, inguinal groin, and left ventricle (**Figures 2A, B**). The nasopharyngeal roof and bilateral walls were symmetrically swollen, with increased metabolism and a tendency to inflammation (**Figure 2A**, 2021-02-05). The patient completed 3-cycle chemotherapies on 2021-03-03, 2021-03-26, and 2021-04-19, respectively. We administered the standard chemotherapy regimen for primary squamous lung cancer: paclitaxel combined with DDP. The primary lesion was markedly reduced in size after two cycles of chemotherapy. The oncological control was satisfied at this point (**Figure 2C**). At the same time, the patient received concurrent radiotherapy for the lumbar spine, inguinal metastatic lymph nodes, and primary lung lesions. The detailed radiotherapy regimens for each site were shown in **Figure 3** and **Table 1**. After radiotherapy, his low back pain and cough symptoms were relieved. The patient was re-evaluated following radiotherapy and 3-cycle of chemotherapies. The imaging showed stabilized lesions of pulmonary origin, appendix, and inguinal metastases (**Figures 2D, E**). Unfortunately, after cycle 3 of treatment, the patient had progressive enlargement of the metastases at the top of the skull and new metastases in the nasal cavity (**Figure 2F**). We recommend that this patient receive chemotherapy combined with Carelizumab (anti-PD-1) immunotherapy on 2021-05-20. Unfortunately, the patients died four months later.

**Table 1:** Timing and plan of radiotherapy.

Site of radiotherapy	Start time	End time	GTV^1^	GTVn^2^	CTV^3^
Lumbar spine	2021-02-24	2021-03-09	35Gy/10F	/	30Gy/10F
Inguinal metastatic lymph nodes	2021-03-31	2021-04-30	52Gy/23F	/	/
Primary lung lesions	2021-04-15	2021-05-11	47.6Gy/19F	41.8Gy/19F	/

1. GTV, Gross tumor volume; 2. GTVn, Lymph node target volume ; 3. CTV, Clinical target volume.

## Discussion

Cecum metastases from squamous lung cancer are extremely rare. This case reports a rare case of squamous lung cancer metastasis to the appendix, scalp, and bone. Early examination confirmed the presence of cecum metastasis without clinical symptoms in this case. The vast majority of patients with LUSC have no clinical manifestations (23). LUSC usually requires ancillary testing and pathological biopsy to confirm (24–26). This patient’s aggressive evaluation at the onset of symptoms was consistent with our care principles. Unfortunately, the patient had multiple metastases and no indication for surgery at the diagnosis. Therefore, we could only use radiation therapy to control the disease.

In this study, we gave the patient the standard chemotherapy regimen for primary squamous lung cancer: paclitaxel combined with cisplatin. After several cycles of chemotherapy and radiotherapy, the disease was controlled. However, the lesion soon became uncontrolled again and worsened. This phenomenon suggests that patients with squamous cell carcinoma of the lung that metastasizes to the appendix have a poorer prognosis with standard radiotherapy regimens (24, 27). More processing methods and optimization techniques still need to be further explored.

## Clinical features of LUSC with gastrointestinal metastasis

Metastasis to the gastrointestinal tract secondary to lung cancer is considerably frequent in necropsy series (14%) (28), but is seldom recognized clinically. It is a rare disease, with a low incidence of 0.2% to 1.8% in clinical studies (8, 9, 29). The common sites are the small intestine and colon (24). Therefore, the appearance of cecum metastasis is very unusual. Detection of gastrointestinal tract abnormalities is usually incidental during the diagnosis of primary lung cancer; occasionally, these abnormalities are detected even earlier than lung cancer. Diagnosis usually can be made using computed tomography (CT), magnetic resonance imaging (MRI), bone scans, pathological biopsies, and endoscopic and immunochemistry studies. Most patients are found to have gastrointestinal metastasis on imaging at the time of diagnosis but with few clinical symptoms in the intestinal tract. Some patients with gastrointestinal metastasis of LUSC have intestinal symptoms, mostly in the form of abdominal pain, gastrointestinal bleeding, perforation, obstruction, constipation, and other symptoms (30–35). In **Table 2**, we summarize previously reported cases of gastrointestinal tract metastasis from primary LUSC with intestinal symptoms.

**Table 2:** The previously reported lung squamous cell carcinoma with gastrointestinal metastasis.

Author/Year	Age/Sex	Digestive tract symptoms	Transfer site	Transfer Time	Maximum diameter of metastatic tumor	Treatment of primary tumors	Treatment of metastatic tumors in the gastrointestinal tract
Nakamura, T., et al. (2015)	88/F^1^	abdominal pain, vomiting, black stool	ileum	12 years	4 cm	surgery	surgery + chemoradiotherapy
Shiraishi, T., et al. (2020)	85/M^2^	fever, abdominal pain	vermiform appendix	unknown	8 cm	surgery	surgery
Uner, A., et al. (2005)	58/M	intestinal obstruction, abdominal pain	colon	19 months	8 cm	surgery	surgery
Yamada, H., et al. (2011)	66/M	ventosity	duodenum, small intestine	coincidence	5 cm	chemoradiotherapy	surgery
Cedrés, S., et al. (2012)	81/M	abdominal pain	rectum	3 months	3 cm	chemoradiotherapy	radiotherapy
Lu, T., et al. (2021)	75/M	emesis, ventosity, icterus	pancreas	1 year	4 cm	surgery + chemoradiotherapy	surgery
Lu, T., et al. (2021)	67/M	emesis, abdominal pain	pancreas	3 years	4.6 cm	surgery	surgery + chemoradiotherapy
Li, X., et al. (2018)	61/M	ventosity	stomach, small intestine	coincidence	0.8 cm/3 cm	chemoradiotherapy	surgery
Moazzam, N., et al. (2002)	54/M	asitia, abdominal pain, icterus	pancreas, biliary tract	coincidence	unknown	chemotherapy	chemotherapy
Ishikawa, T., et al. (2017)	70/M	abdominal pain	pancreas	coincidence	3.8 cm	chemotherapy	chemotherapy
Stoupis, I., et al. (2020)	60/F	asitia	pancreas	coincidence	4.5 cm	immunotherapy	immunotherapy
He, Y., et al. (2019)	61/M	dysphagia	stomach	coincidence	4 cm	surgery + chemotherapy	surgery
Sakai, H., et al. (2012)	60/M	abdominal pain	colon	6 months	unknown	surgery + chemotherapy	chemoradiotherapy
Papaziogas, B., et al. (2012)	68/M	abdominal pain, emesis	small intestine	8 months	4 cm	surgery	chemotherapy
Liu, W., et al. (2015)	66/M	black stool	small intestine	4 months	4.5 cm	surgery + chemotherapy	surgery
Carroll, D., et al. (2001)	68/M	diarrhea	colon	coincidence	4 cm	chemotherapy	surgery
Nemoto, M., et al. (2020)	64/M	dysphagia	stomach	1 year	5 cm	surgery	surgery
Tanaka, T., et al. (2011)	85/M	abdominal pain	jejunum	10 years	unknown	radiotherapy	surgery
Zhou, W., et al. (2020).	63/M	abdominal pain, icterus	pancreas	6 months	4.5 cm	surgery	surgery
Hirasaki, S., et al. (2008).	74/M	center lower abdominal tenderness	colon	coincidence	4 cm	chemotherapy	chemotherapy
Wang, W., et al. (2017).	61/M	abdominal pain, emesis	stomach	coincidence	unknown	surgery	surgery
Jeong, Y., et al. (2012).	79/M	abdominal pain, emesis	bile cyst	coincidence	3.5 cm	alleviative treatment	alleviative treatment
Miyazaki, J., et al. (2015).	54/M	abdominal pain, anemia	stomach, colon, caecum	unknown	unknown	surgery + chemotherapy	chemotherapy
Weiss, G., et al. (2013).	67/M	astriction	colon	coincidence	unknown	chemotherapy + targeted therapy	chemotherapy + targeted therapy
Weiss, G., et al. (2013).	60/M	astriction	sigmoid colon	coincidence	unknown	unknown	unknown
Memon, Z., et al. (2017).	81/M	black color stool	duodenum	coincidence	unknown	palliative chemoradiotherapy	palliative chemoradiotherapy
Kadowaki, T., et al. (2005).	72/M	abdominal pain	liver	2 years	unknown	surgery	alleviative treatment
Kyriazi, M., et al. (2009).	77/M	abdominal pain, emesis, icterus	pancreas	2 years	unknown	surgery + chemotherapy	surgery
Meneses Grasa, Z., et al. (2009).	69/M	abdominal pain, emesis	mesentery	6 months	15 cm	surgery + chemotherapy	surgery + chemotherapy
Bhardwaj, R., et al. (2017).	39/M	black color stool	stomach	6 weeks	6 cm	chemotherapy	radiotherapy
Lou, H., et al. (2014).	64/M	abdominal pain	colon	3 years	8.5 cm	surgery	chemotherapy
Azar, I., et al. (2017).	90/M	astriction	liver, stomach	2 months	8 mm/unknown	none	none
Machairas, N., et al. (2019).	78/M	icterus	pancreas	3 years	2.5 cm	surgery + chemotherapy	surgery
Win, A. and C.J.J.o.c.i.s. Aparici (2015)	68/M	asymptomatic	rectum, liver	coincidence	unknown	palliative treatment	palliative treatment

1. F, Female; 2. M, Male.

## Diagnosis of LUSC with gastrointestinal metastasis

LUSC is prevalent in clinical practice, and its clinicopathological features are remarkably clear. Squamous cells are “scaly structures” that occur along the trachea and bronchi. Pathologists categorize squamous cells by “keratin pearls” under a microscope. LUSC immunophenotypes consistently express P63 and are negative for TTF1 (36). Other squamous immunomarkers include CK5/6 or P40. Several clinical studies have shown that squamous cell lung cancer is more likely to metastasize to the gastrointestinal tract than other lung cancers (37, 38). Therefore, whether the histological types are associated with gastrointestinal metastases remains unknown. The correct diagnosis of pulmonary tumors is crucial for treatment decisions. Clinically, the origin of the tissue type is often identified by immunohistochemistry (25). Information on the expression of immunohistochemical markers facilitates histopathological diagnostics. In most patients with LUSC, the expression of immune markers in metastatic sites was consistent with that in the corresponding primary tumors. Immunoprotein staining is consistent between the tumor’s gastrointestinal and pulmonary origin, and positive staining for CK-14 and CK-18 suggests squamous cell carcinoma and adenocarcinoma (26). Gastrointestinal metastasis from LUSC is rare and unique in clinical practice and is sometimes mistaken for primary digestive tract tumors (24, 30). Thus, some ancillary examinations help provide the basis for the diagnosis.

Laboratory examination, endoscopy, gastroenterography, CT, and positron emission tomography (PET)-CT may aid in diagnosing LUSC in patients with gastrointestinal metastases (24). CT is the mainstay for noninvasive diagnosis and staging of many gastrointestinal tumors, its positive signs can be recognized in gastrointestinal metastases patients, including localized gastrointestinal wall thickening, the presence of a mass in the gastrointestinal cavity, intussusception, and perforation (23). Abdominal pain is the most common clinical symptom of these patients. However, the symptoms are not entirely consistent with the severity of illness in some clinical settings. Most patients are no clinical symptoms. Consequently, most gastrointestinal metastases are associated with intestinal perforation, intractable gastrointestinal bleeding, intestinal obstruction, and other serious complications, thereby leading to accidental death. Endoscopic biopsy provides an opportunity for diagnosing unexplained metastases and for treatment, especially in patients with gastrointestinal bleeding. Endoscopy is more accurate than CT and MRI because it can depict small lesions that other imaging modalities cannot. In addition, PET-CT has good sensitivity and specificity for detecting metastatic tumors and is commonly used in diagnosing metastatic tumors of the gastrointestinal tract (39–41). However, pathology remains a critical factor for the final diagnosis of LUSC with intestinal metastasis. Simultaneously, the pathology of primary lung cancer should be compared with that of metastatic lesions in the gastrointestinal tract, which is crucial for patients.

## Treatment of LUSC with gastrointestinal metastasis

Although the treatment patterns are rapidly changing, treatment options for first-line therapy of advanced LUSC remain limited compared to those for other types of lung cancer. Most patients with advanced LUSC have good survival after radiotherapy and chemotherapy. Platinum-based chemotherapy regimens have been shown to improve survival and enhance patient quality of life. However, rare cases with gastrointestinal metastasis have a poor prognosis with a median overall survival of only 4–8 weeks (24, 27, 30). Thus, LUSC patients with gastrointestinal metastasis should undergo early aggressive surgical treatment or local ablative therapy (26). Both of these are suitable for patients with good performance status. Resection of isolated gastrointestinal metastasis has been shown to improve the survival of patients with LUSC. Compared to surgical treatment (lobectomy for primary pulmonary tumors and lymphadenectomy and endarterectomy), local ablative therapy is less invasive, more beneficial, and recommended for patients.

Distinguishing squamous cell carcinoma from adenocarcinoma is vital for drug selection. Unlike lung adenocarcinoma, squamous cell lung cancer lacks effective targets, including mutations and alterations, for which the approved targeted treatments are rare in LUSC (42–47). Consequently, it is critical that the use of new treatment modalities be taken into account to ensure that patients with LUSC receive the most appropriate treatment and have better outcomes. Given the approval for targeted therapies and immunotherapies for advanced NSCLC and the extension toward personalization of advanced lung cancer treatment, these methods can also be applied to patients with LUSC with gastrointestinal metastases to achieve better outcomes.

## Conclusions

Treatment of advanced LUSC remains challenging because of specific tumor characteristics. These characteristics result in fewer treatment options and shorter overall survival. Herein, we report the treatment process of a rare case of squamous lung cancer with metastases to the cecum, scalp, and bone. Cecum metastasis is rare in LUSC. This case demonstrates the poor prognosis of squamous lung cancer metastasizing to secondary lymphoid organs of the gastrointestinal tract. Therefore, patients with squamous cell carcinoma of the lung who develop gastrointestinal metastases are advised to prolong their survival through surgical resection or local ablative therapy once detected.

## Data availability statement

The original contributions presented in the study are included in the article/supplementary material. Further inquiries can be directed to the corresponding authors.

## Ethics statement

Written informed consent was obtained from the individual(s) for the publication of any potentially identifiable images or data included in this article.

## Author contributions

HW, QL, FFL designed, discussed, wrote, and submitted this manuscript. YQL, KX, QY access to literature. All authors contributed to the article and approved the submitted version.
